# Review of Perioperative Music Medicine: Mechanisms of Pain and Stress Reduction Around Surgery

**DOI:** 10.3389/fmed.2022.821022

**Published:** 2022-02-04

**Authors:** J. P. Ginsberg, Karthik Raghunathan, Gabriel Bassi, Luis Ulloa

**Affiliations:** ^1^Departments of Applied Psychophysiology, Psychology and Statistics, Saybrook University, Pasadena, CA, United States; ^2^Critical Care and Perioperative Population Health Research Unit, Department of Anesthesiology, Duke University Medical Center, Durham, NC, United States; ^3^Department of Anesthesiology, Center for Perioperative Organ Protection, Duke University Medical Center, Durham, NC, United States

**Keywords:** perioperative, music medicine, stress, Heart Rate Variability, autonomic nervous system, vagotomy, review

## Abstract

Clinical-experimental considerations and an approach to understanding the autonomic basis of improved surgical outcomes using Perioperative Music Medicine (PMM) are reviewed. Combined surgical, psycho-physiological, and experimental perspectives on Music Medicine (MM) and its relationship to autonomic nervous system (ANS) function are discussed. Considerations are given to the inter-related perioperative effects of MM on ANS, pain, and underlying vagal and other neural circuits involved in emotional regulation and dysregulation. Many surgical procedures are associated with significant pain, which is routinely treated with post-operative opioid medications, which cause detrimental side effects and delay recovery. Surgical trauma shifts the sympathetic ANS to a sustained activation impairing physiological homeostasis and causing psychological stress, as well as metabolic and immune dysfunction that contribute to postoperative mortality and morbidity. In this article, we propose a plan to operationalize the study of mechanisms mediating the effects of MM in perioperative settings of orthopedic surgery. These studies will be critical for the implementation of PMM as a routine clinical practice and to determine the potential limitations of MM in specific cohorts of patients and how to improve the treatment.

## Clinical Benefits of Perioperative Music Medicine

Surgery is an “indivisible, indispensable part of health care” around the world (1, 2) but it causes significant physiologic stress (3, 4). Neural (autonomic) and humoral (circulating or hormonal) mechanisms primarily involve the sympathetic nervous system (SNS) adrenergic and parasympathetic nervous system (PNS) cholinergic branches of the autonomic nervous system (ANS). Current perioperative interventions used to attenuate these stress responses are primarily pharmacologic (anesthetic and analgesic medications) that are associated with a variety of complications (5). The current epidemic of opioid overuse in the United States is fueled in part by excessive perioperative prescriptions around both minor and major surgery (6, 7). Non-pharmacologic interventions that complement and integrate with pharmacologic interventions in the perioperative setting are thus of critical importance to both decrease psychophysiological stress *and* decrease opioid use. Perioperative Music Medicine (PMM), defined as listening to pre-recorded music around surgery (8), is an efficacious, safe, and low-cost non-pharmacologic intervention that can be delivered at the point-of-care and reduce opioid use (9–11).

## Perioperative Music Medicine Translational Research Continuum

**Figure 1:**
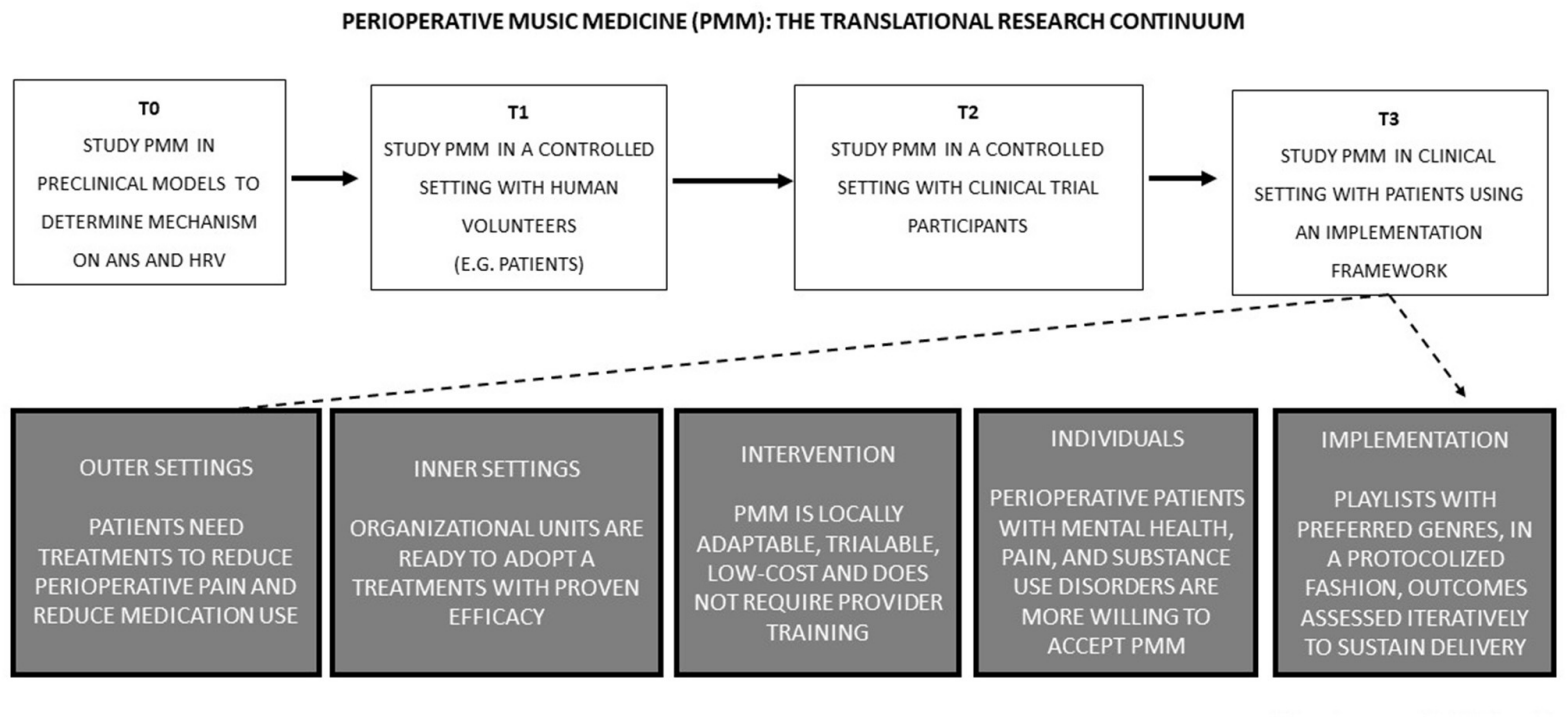
Perioperative Music Medicine (PMM) translational research continuum. Schematic diagram of translational research on PMM for the implementation of new more effective treatments based on mechanistic studies.

**Figure 2:**
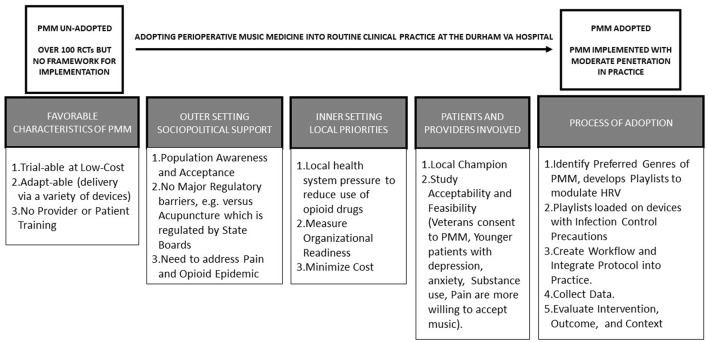
The evidence to practice gap in implementing PMM practice. Schematic representation of the translational strategy from evidence to practice to implement the use PMM into routine clinical practice in orthopedic surgery at the Durham VA Hospital.

While the specific mechanisms of PMM remain unclear, preclinical (T0) studies suggest that the auditory pathway must be intact for an effect (Figure 1). In rodent models, physiological effects include a shift from sympathetic toward more parasympathetic autonomic activity, suppression of stress hormones, lowering heart rate, blood pressure, and anxiety, but increasing immune functions (12). In human volunteers (T1 studies) and clinical trial participants (T2 studies), there is a strong body of evidence supporting the efficacy of PMM when compared to ambient noise, ear plugs, or headphones with noise cancellation or white noise (9–11). This translational strategy has been critical in implementing the use of PMM at the Durham VA hospital for the treatment of veterans in North Carolina (Figure 2). In recent meta-analyses of randomized controlled trials (RCTs), PMM was estimated to cause clinically important reduction in pain and anxiety (~10 and 20 mm, respectively, on the 100 mm visual analog scale) (9–11), and decreased opioid use (~10 mg lower in morphine equivalents over three postoperative days) (11) (Figure 2). In this section, we briefly review the clinical benefits of PMM on cognitive and affective pathways, as well as the favorable modulation of the ANS and implementation.

### PMM Acts on Cognitive and Affective Pathways

Noise contributes to adverse health outcomes in various settings, and it is a significant stressor in operating rooms (ORs) with an average intensity between 51 to 75 dB (13). Highest noise levels are found in the ORs in which orthopedic surgery is performed (14). Ear plugs and noise canceling headphones can therefore have benefits, especially around joint replacement surgery (13). Musical activation of the auditory system has been shown to inhibit pain pathways beyond noise cancellation effects (15). Consistent with modern theories of pain (16), PMM has cognitive effects impacting both intensity and quality of pain (9–11), and PMM also lowers anxiety (on average by 5.72 units vs. standard care, when measured by the State Trait Anxiety Index) (17) in both adult and pediatric populations (18). Perioperative Music Medicine decreased pinprick pain, repeated (conditioned) pain stimulus, and also increased the threshold for pressure pain (19). As a distracting stimulus it shifts attention away from competing noxious stimuli (20). Perioperative Music Medicine is especially effective in patients with depression (21), increasing emotional activity centered in the limbic system of the brain which feeds into the common pain pathways (18), produces endogenous opioids (18) and oxytocin (18, 22) as well as decreases cortisol (22) and catecholamines (22, 23). Thus, PMM may work well in the awake patient. Benefits may be greater when PMM is self-selected (24), because the way in which patients cognitively engage with the music is different when patients choose the type of music.

### PMM Acts on the Autonomic Nervous System

There is a large body of research on the effects of music on the ANS in healthy subjects, and findings include significant reductions in heart rate (especially when listening to soft music) (25) in *both* conscious and unconscious volunteers (26). There is now strong evidence that hearing is perceptive during general anesthesia (27), and auditory stimuli—including music under general anesthesia—could modulate the neurohormonal response to surgery. Cortical auditory evoked responses are not abolished by inhalational anesthetics at the concentrations used for surgery (28). This provides a basis to understand the clinical studies where PMM *under general anesthesia* decreased postoperative pain, analgesic use, anxiety, respiratory rate, blood pressure, cortisol levels, postoperative nausea, and vomiting (9–11, 29).

How Heart Rate Variability (HRV) is measured and what it represents is discussed in the next section of this article, but evidence regarding the effects of PMM on HRV is inconsistent (25). While it is well-established that HRV decreases during anesthesia (30, 31) and that decreased HRV is associated with greater morbidity and mortality (32), it is not known whether PMM has clinical benefits when changes in HRV are invoked. Since HRV around surgery may aid in evaluation of the perioperative nociception-analgesia balance in both adults and children (32), the effects of PMM may be viewed through the lens of changes in HRV. In other words, the causal pathway from PMM under general or spinal anesthesia to clinical benefits may go through modulation of ANS activity as reflected in changes in the HRV. The last section proposes an experimental setup where HRV is measured throughout the perioperative experience (under general or spinal anesthesia during joint replacement surgery) with subjects randomly assigned to receive or not PMM. We hypothesize that clinical benefits (less pain, anxiety, and medication use) of PMM will depend on its potential to increase the vagal tone and HRV. Our hypothesis is that a decreased HRV could signal a surgery-induced sympathetic stress whereas an increased HRV could signal a parasympathetic resilience to surgical stress. Our hypothesis is that benefits of PMM on surgical outcomes are accompanied by increased HRV, indicating that the effect resides in resilience to surgical trauma due to reduced stress responses.

### PMM Can Be Implemented at the Point-of-Care

For patients to broadly experience the clinical benefits of PMM, it has to be adopted with fidelity and become a part of the local “culture.” We recently conducted (T3) research to translate evidence (9–11) into routine delivery of PMM to patients in our daily clinical practice (33). Implementation of PMM was accelerated by the need to respond to the opioid epidemic, and by the relative advantage it offered over other non-pharmacologic interventions including mindfulness, biofeedback, acupuncture, Yoga, and Tai Chi (33). Perioperative Music Medicine can be adapted to the local context and the protocol for PMM can be refined in iterative cycles on a small-scale. Provider credentialing is not required (as in the case of acupuncture) and patient training is also not required (as in biofeedback, Yoga, and Tai Chi). After assessing organizational readiness for implementation and identifying local champions (34, 35), the capital investment to deploy PMM is modest with the development of playlists based on local patient preferences, purchase of digital music players and covers (for infection control), and extracting data already being collected for clinical purposes (pain scores, medication use, satisfaction scores). Perioperative Music Medicine was welcomed as an exciting complement to opioid and non-opioid analgesia in the perioperative period, and is currently sustained with moderate penetration (such that over two-thirds of the patients who want to receive this intervention actually get it) (33).

Further research is warranted because a path to delivery at the bedside already exists. In the next two sections we review ANS responding to psychophysiological stress and HRV analyses, and the last section proposes combining perioperative clinical with a controlled experimental approach to uncover the mechanisms by which PMM attenuates stress and improves surgical outcomes.

## Music Medicine and the ANS

Whereas, Music Therapy (MT) is primarily a cognitive rehabilitation method involving a therapist (36–38), Music Medicine (MM) is the delivery of prerecorded music through headphones, musical pillows, or background sound systems as a treatment for clinical disorders, including peri-operative surgical procedures. In our view, music is the active component that accounts for the effectiveness of both approaches, and the two procedures share much in common for disease management. Meta-analyses of RCTs of MM and MT (vs. either no music, white noise, undisturbed bed rest, or usual care in various pain conditions) found that the reduction in pain, anxiety, and opioid use exceeded minimum clinically important differences (39, 40). Surprisingly, and despite the evidence, MM is not widely used. We propose that the central mechanism underlying the benefit of PMM, and MM in general, is the enhancing effect the intervention has on emotional regulation mediated through ANS homeostasis, which can be indexed by HRV. Our overall hypothesis is that MM reduces clinical morbidity by decreasing sympathetic activation and preserving homeostasis of stress metabolism and immune function *via* strengthening parasympathetic cardiac cholinergic output from the myelinated neurons in the nucleus ambiguous of the vagus nerve. This strengthening of parasympathetic cardiac cholinergic output (“vagal tone”) can be indexed quantitatively by changes in HRV.

### Autonomic Nervous System Stress and Heart Rate Variability

Under normal stress, ANS cardiac control reflects an adaptive level of interplay between the PNS and SNS. The PNS produces cardiac deceleration (“rest and digest”) *via* myelinated vagal nerve (CNX) cholinergic output from nucleus ambiguous (also referred to as the “ventral vagal complex”) (41) onto the heart. Acetylcholine (ACh) released by ventral vagal stimulation reduces heart rate by activating the M2 muscarinic receptors (M2R) that, in turn, opens the ACh-activated potassium channel to slow the firing of the pacemaker cells in the sinoatrial (SA) node (42, 43). The right vagus nerve primarily innervates the SA node and slows its pacemaker whereas the left vagus innervates the AV (atrioventricular) node and slows its conduction of the cardiac impulse to the bundle, but the ventricular myocardium is sparsely innervated by vagal efferent signals (44). The SNS produces cardiac acceleration (“fight or flight”) as the individual orients in the social and physical environment (45). Sympathetic efferent nerve endings are present in the SA node and throughout the atria and ventricles (44). In simplified terms, the interplay of the two branches of the ANS allows adaptation to normal stress in the face of everyday challenges by SNS signaling to hypothalamic-pituitary-adrenal (HPA) axis leading to release of corticosteroids (cortisol in humans, corticosterone in rodents) and pro-inflammatory factors from the adrenal gland (46, 47). Adrenal glucocorticoid release is regulated by a negative feedback signal to hippocampal glucocorticoid receptors that shuts off stress responding and the corticosteroid and pro-inflammatory neuromodulators when the stress ends (48).

As previously mentioned, the status of dynamic sympatho-vagal ANS function can be indexed in real time with HRV (49). Heart Rate Variability is basically defined as quantitative analysis of variation in the time interval between heartbeats (interbeat interval, IBI). Heart Rate Variability is an indicator of the magnitude and pattern of changes in inter-beat intervals. The change in successive IBI's (standard deviation of the normal-to-normal pulse, SDNN) in a normal individual is typically in the range of 40–60 ms (50). Thus, HRV can be measured so long as the pulse recording device can sample at a rate ≥1,000 Hz (which corresponds to measurement of a change in IBI of 1 ms). A recording of IBI's can be reported with a number of different variables (49). Time domain variables are direct calculations based on IBI's; frequency domain variables are produced by a Fast Fourier Transform of the IBI time series to yield a power spectrum. In general, lower HRV values indicate SNS dominance while higher HRV values indicate a shift to PNS dominance (49).

However, the simple definition of HRV as the pattern of variation between consecutive heartbeats belies the complexity, meaning, and significance of the many different measures of HRV (51) and so there is a potential for *apophenia*—incorrect conclusions or excessive, unfounded extrapolation (52)—that must be guarded against. Nonetheless, carefully applied quantitative analysis of IBI data can be used to index fundamental systemic clinical physiological processes (53). Because there are numerous methods of analyzing IBI data as various HRV indices [time-domain, frequency-domain, geometric, non-linear, and fractal/complexity (49, 50, 54, 55)], caution must be taken in the selection of the HRV analyses used in any particular context.

Healthy HRV contains a regular pattern of increasing and decreasing IBIs between consecutive beats that increases HRV, while unhealthy HRV is relatively low when little variation between IBIs or random, unorganized differences between consecutive beats exists (50). The rhythm of cardiac acceleration–deceleration is linked to inhalation–exhalation of the respiratory cycle (inhale, cardiac acceleration; exhale, cardiac deceleration), called respiratory sinus arrhythmia (RSA). The regular respiratory cycle-related of increasing and decreasing IBIs approaches a sinusoidal pattern. Slow deep breathing (around six breaths per min) is called resonance frequency breathing because at that frequency the amplitude of highest to lowest IBI over the respiratory cycle reaches a maximum due to inputs from the baro-receptor and vasomotor reflex maintaining blood pressure homeostasis. Low HRV is the single most accurate clinical predictor of mortality after cardiac events, especially in the elderly (56–59). Current studies reveal that relatively low HRV is also related to several chronic physical illnesses (60), stress (61), and certain forms of mental disorder (62–65), while relatively high HRV is related to optimal physical (66, 67), and cognitive (68–70) performance.

### Chronic Pain, Chronic Stress, and HRV Are Inter-related

The sensation of acute nociceptive pain is due to injury to peripheral sub-dermal pain fiber endings, primarily C and Aδ fibers (71). C fiber pain transmission is mediated by the ANS (72). When acute nociceptive pain becomes chronic, central pain sensitization often occurs (73). Chronic HPA stress responding due to chronic nociceptive pain interacts with ANS cardiac control and produces persistent sympathetic activation (74). When central pain sensitization occurs, previously sub-threshold synaptic inputs to nociceptive neurons augment action potential output and thus chronic sensitized pain reflects additional contribution from dysregulated CNS and ANS function to the experience of pain (75). However, it is important to note that nociceptive pain that persists after resolution of the initial insult to nociceptive pain fibers cannot be said to be entirely uncoupled from the nociceptive receptors. Sensitized chronic pain states in which tissue inflammation or pathology is not readily apparent (for example, fibromyalgia, irritable bowel syndrome, painful bladder syndrome, or migraine) occur despite the absence of a pathobiological explanation, implicating unresolved nociceptor sensitization in addition to CNS and ANS contributions (76).

Stress from continual pain creates persistent hyperarousal of SNS, known as HPA overdrive (77–79). Hypothalamic-pituitary-adrenal overdrive, such as also arises from social and environmental chronic stress, increases glucocorticoid signaling from the adrenal cortex that deregulates the negative feedback signal to shut off stress, down-regulates hippocampal glucocorticoid receptor expression, and ends the normal negative feedback tampering the stress response (80). Chronic sensitized pain is also associated with inhibition of descending cortical pain modulation by periaqueductal gray, rostroventromedial medulla, and serotonergic and adregeneric euromodulators (“nociceptive braking”), producing further proliferation of peripheral pro-inflammatory cytokines (81). Thus, pain with chronic stress responding becomes a self-reinforcing cycle, causing the pain threshold to be lowered, and a neuro-modulator picture that is equivalent to depression (77, 78, 82–84). Pain is thus an internal stressor that first triggers a long-term tonic response of the hypothalamic-pituitary adrenal system when pain becomes chronic. Physiological activation leads to both persistent sympathetic arousal lowering HRV (50). Numerous studies have confirmed that HRV variables are lowered by chronic pain (85–94). In addition to chronic stress from chronic pain signals that originate in nociceptive nerve fiber lesions, psychological catastrophizing, and unremitting fear rumination further augment the prolonged stress response and are core aspects of chronic pain sensitization (95, 96).

### HRV, Chronic Pain, and Chronic Stress Are Associated With Inflammation, Depression, and Anxiety

Inflammation is commonly known to accompany the stress reaction, and the association of HRV with persistent sympathetic arousal typical of stress responding has been well-characterized (74, 77). There are several studies showing that HRV and inflammation consistently have an inverse relationship, depicting that inflammation is associated with SNS arousal (97–104).

**Figure 3:**
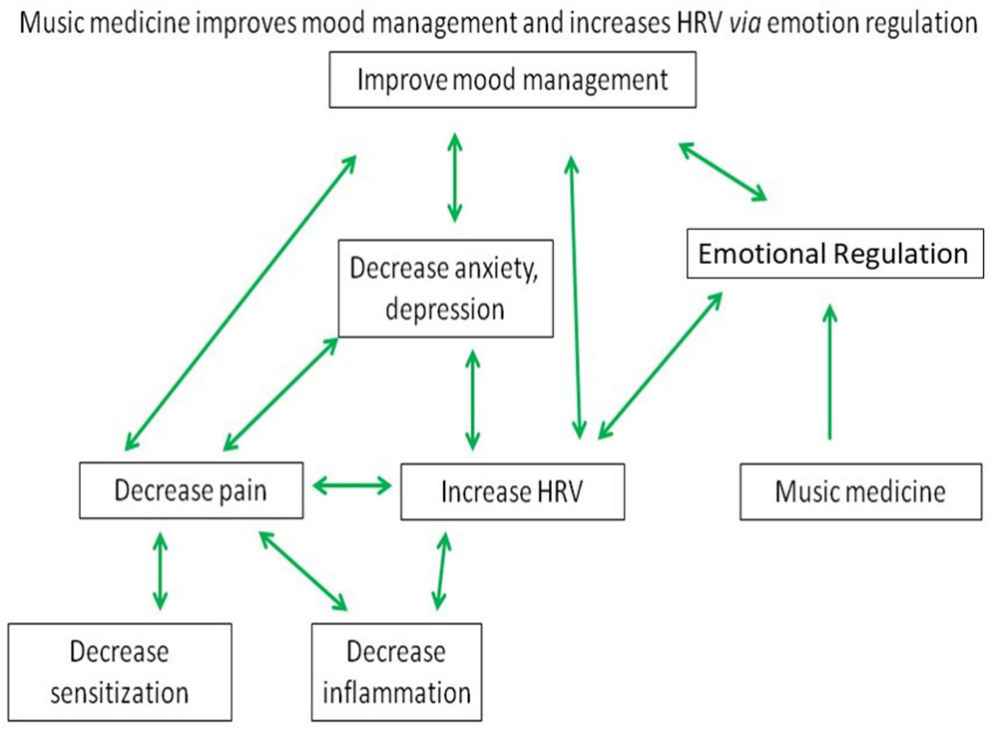
Multifactorial regulation of HRV by music medicine. Proposed Model for Music Medicine to improve mood management and HRV *via* emotional regulation integrating the regulation of pain, inflammation, anxiety, and depression.

The observation of decreased HRV associated with depression and anxiety has been widely reported in the literature (105–109) (Figure 3). Findings of increased occurrence of cardio-vascular diseases among patients with Major Depressive Disorder have drawn attention to autonomic regulation of the heart rate as a potential pathophysiological mechanism in depression (110). Dysregulation of autonomic cardiac control resulting in lowered HRV is clearly associated with anxiety and traumatic stress (111, 112). State, trait, and clinical expressions of anxiety are considered a restricted response range across biological and behavioral functioning reflecting diminished vagal tone and thereby HRV (62). Thus, chronic pain is readily observable by lowered HRV values because chronic pain, chronic stress, and HRV are inter-related and associated with catecholamines (113), inflammatory mediators (114), depression, and anxiety (77, 115).

### Many Different Procedures Increase HRV

Rhythmic stimulation around the 0.1 Hz frequency from a surprising array of different sources have been shown to have cardiovascular (CV) effects. Rhythmic stimulation that increases HRV includes: instrumental autotraining (116), paced breathing (HRVB) (49, 117–120), emotionally salient pictures (121), chanting and toning (122, 123), prayer and yoga mantra (124), poetry (125–127), skeletal muscle tension (128, 129), orthostatic tilt (130, 131), thermal stimulation (132), and neck suction (133). Perhaps the earliest published study of increased HRV due to biofeedback was authored by Vaschillo et al. (116), who observed that Russian cosmonauts could control their breathing while viewing a computer display of their breathing rhythm and heart rate oscillations until their breathing became synchronized with their heart rate oscillations. Although Vaschillo termed this process “instrumental autotraining,” the cosmonauts were engaging in a form of biofeedback by controlling their breath cycle to match a computer screen-presented display of heart rate oscillation. Paced slow breathing is very effective at increasing HRV (134–137) and appears to be the most widely used method for increasing heart rate oscillations and associated HRV (138, 139). Current techniques of HRV biofeedback (HRVB) are based on coaching (140) along with the use of a breath pacer (140) in addition to visual display feedback of heart rate oscillation and respiratory cycles.

Increased heart rate oscillations due to paced slow breathing, which is trained in HRVB results from synchronization, or phase-locking, of interacting cycles among physiological systems controlling respiration, heart rate, arterial blood pressure, and vasomotor tone, *which* increase heart rate oscillations and HRV primarily through the baroreceptor reflex (128, 141–143). As a result, the maximum to minimum IBI during a single breath cycle—a basic definition of HRV (50)—is amplified. Vagal afferent signaling from heart rate to cortex (141, 144–147) and a neural pathway based on delta oscillation reduction from prefrontal cortex to vagus (148) influencing vagal efferent signals back to the heart are also recognized.

Increased HRV reflects dampened HPA hyperarousal, calming of the SNS, stimulation of PNS activity, and lowered heart rate, resulting in reduced stress responding. The understanding that heart rate reflects bidirectional heart-brain processes involved in emotional arousal is hardly recent and was put forth by early modern life scientists French physiologist Claude Bernard in 1867 (149) and Charles Darwin in 1872 (150). The relationship of HRV to environmental response and adaptation recently has been re-affirmed (140):

“… the complex mix of physiological, behavioral, emotional, and cognitive processes involved in self-regulation and adaptability might have a common basis such that indices of HRV would be associated with all of these various forms of regulation (p. 82). We have proposed that the relationship between HRV and important physiological, cognitive, and emotional regulation functions is due to the ability of HRV to index activity in a flexible network of neural structures that is dynamically organized in response to environmental challenges.” (p. 86)

Underscoring this relationship between HRV and improved adaptation and stress responding is evidence that a number of psychotherapies that are based on self-regulation of thoughts and emotions that improve well-being—such as Polyvagal-informed Therapy (151, 152), Compassion Meditation (153), Mindfulness (154–156), Acceptance and Commitment (157), Cognitive Behavior Therapy (158), Forgiveness (159), and Creative Arts (160)—when applied without incorporation of additional training in slow paced breathing, have also been shown to be accompanied by increases in HRV.

### Emotional Regulation Is Improved by Increased HRV

Emotional regulation is the ability to exert control over one's own emotional state, for example controlling thoughts during a challenging situation to reduce anger or anxiety, managing signs of sadness or fear, or focusing on reasons to feel happy or calm. Emotional regulation involves self-monitoring, initiation, maintenance, and modulation of positive and negative emotions, and the avoidance and reduction of high levels of negative affect (161). The function of emotional regulation is adaptation produced by interaction between biological constraints and the physical and social environments. Adaptive emotional regulation is sometimes mistakenly equated with minimization of negative emotion. Healthy emotional regulation is based on flexible but stable behavior patterns of individuals, so both positive and negative emotions are experienced and expressed with a level of intensity that is matched to events, with the goal of achieving successful social interactions and safety.

Perhaps the earliest published study showing that increased HRV due to an intervention using HRVB was beneficial for people with psychological disorder was also authored by Vaschillo et al. (162). HRVB, currently the most widely used technique to increase HRV, is established as a reliable and effective way to improve emotional well-being, mental acuity, and physical function (105, 163). HRVB paced breathing is used for clinical intervention of psychological disorder both with and without accompanying “top-down” emotional regulation coaching (120, 164). Inducing high amplitude heart rate oscillations *via* slow paced breathing without intentional emotional regulation nonetheless enhances the emotional regulation neural network function. By manipulating HR oscillations in isolation, Mather (165) demonstrated that an increase in activation occurred in brain regions associated with emotional regulation after heart rate oscillations increased, but activation in the same regions did not occur after oscillations decreased. Furthermore, a behavioral measure of emotional regulation viewing pictures also increased in the high but not in the low HR oscillation group.

## Music Increases HRV and Improves Emotional Regulation

We propose that PMM exerts a directional influence that restores autonomic homeostasis that can be indexed by HRV and strengthens emotional regulation, thereby leading to improvements in the individual's state of chronic pain, stress, inflammation, anxiety, and depression.

### Music and HRV

Beyond the emotional responses, music affects the CV system and influences HRV (26). A systematic review has confirmed that music, as a stimulus acting on the cardiac ANS, increases parasympathetic activity and HRV. However, there is conflicting evidence on whether it is the music itself or listener preferences that matter and the impact of individualized MT of passive listening vs. preferred soundtracks (25). Optimal music for therapy varies between individuals. Self-selected pieces tend to elicit a eustress or joyous response (“positive arousal”), whereas classical music is associated with the highest HRV for parasympathetic dominance (166).

While several studies have established the potential of music to affect HRV as expected, the relationship between music and HRV is complex; for example, exciting music decreases high frequency (HF) HRV power, which is associated with PNS activation, compared to tranquilizing music (167). Overall, excitative music decreases activation of the PNS (168). For example, auditory stimulation with heavy metal music decreased the sympathetic and parasympathetic modulation on the heart, while exposure to a selected classical baroque music reduced only sympathetic regulation on the heart without affecting the parasympathetic activity (169). Acute exposure to heavy metal music also was shown to increase the sympathetic activity in healthy women (170). Likewise, the low frequency (LF) component of HRV and the LF/HF ratio increased (indicating increased parasympathetic activation) during sad music.

Autonomic responses to musical stimuli were correlated with subjective preferences regarding the relaxing properties of silence, classical, new age, or romantic melodies (171). The LF/HF ratio was significantly higher when subjects were exposed to “new age” music as compared with silence, while no differences were found with “classical” or “romantic” melodies. These results were related to a reduction in the HF component with “new age” as compared to silence. Subjects' preferences did not correlate with autonomic responses to melodies. The results suggest that “new age” music induced a shift in HRV from higher to lower frequencies, independently of the music preference of the listener.

As a preliminary test of our hypothesis that MM positively affects HRV, we performed a calculation of the effect size of music on HRV specifically in perioperative surgical settings (PMM, presented below).

### Music and Emotional Regulation

The effects of music on emotional regulation have been well-studied (172) and music has been shown to be an effective strategy to regulate affective states (173). People employ music to induce specific emotional states in everyday situations for the purpose of emotional regulation, usually expressing preference for pieces of music that were emotionally congruent with an emotional situation (174). Musical characteristics such as tempo and rhythm give can change a listener's mood and emotions (175). Desired emotional activation occurs when listening to preferred and familiar music, while undesired activation patterns arise when introducing complexity, heightened, or unfamiliar dissonance emphasizing the connection between music-influenced changes in attention and a link to emotional regulation (176). Brain activity in emotional regulation using music was studied using psychological testing and functional magnetic resonance imaging (fMRI) (177). In this study, neural responses to music were measured in the medial prefrontal cortex (mPFC) in a cohort of 56 participants. Discharge, that is using music to express negative emotions, lowered medial pre-frontal cortex activity in males and diversion, using music to distract from negative emotions, increased medial pre-frontal activity in females. These findings suggest that using music to discharge negative emotions may be associated with a maladaptive pattern of brain function and have long-term negative effects on mental health.

## Emotional Regulation Is an Important Factor in Chronic Pain, Inflammation, Chronic Stress, and Depression and Anxiety

### Emotional Regulation and Chronic Pain

Maladaptive emotional regulation appears to be a risk factor for the development and maintenance of chronic pain, and is associated with psychological co-morbidities of pain (178). Emotional regulation capacities have been empirically linked to variables of pain coping (179), including opioid abuse (180, 181). In general, emotional regulation mediates the relationship between pain and quality of life (182).

### Emotional Regulation and Chronic Stress

Open and RCTs have demonstrated the utility of emotional regulation therapy in treating stress-related conditions (183). Chronic stress is a risk factor for incident CV diseases and emotional regulation moderates the association between chronic stress and CV disease risk. In a large scale (*n* = 754) study, stress, emotional regulation, and CV risk measures were used to test whether emotional regulation mitigates the effect of chronic stress on CV risk (184). Results showed that stress interacted significantly with difficulties in emotional regulation to affect CV risk. Emotional regulation therapy using a reappraisal strategy alters not only affective components but also brain activity of pathological stress. Reappraisal affects activation of prefrontal cortical areas that reduce activity in limbic areas such as the amygdala, and patients with posttraumatic stress disorder (PTSD) have different brain activity during reappraisal in comparison to individuals without PTSD (185).

### Emotional Regulation and Inflammation

Psychiatric disorders-especially affective disorders including depressive and anxiety disorders are quite common and have been linked to dysfunction in endocrine and immune systems (186). Cytokine signals can access the brain and cause profound changes in neurochemistry, neuroendocrinology, and behavior. For instance, physiological consequences of stressful life experiences derive in part from the effects of stress on the immune response and have relevance for treatment of neuropsychiatric disorders (187). The emotional regulation strategies of reappraisal and suppression were studied in relation to inflammation (C reactive protein, CRP), stress and coronary heart disease in a large cohort of adult offspring from the Collaborative Perinatal Project (188). In this study, the finding emerged that the maladaptive emotional regulation strategy (suppression) was associated with elevated levels of inflammation whereas the adaptive emotional regulation strategy (reappraisal) was associated with lower levels of inflammation. Among couples with marital conflict and poor sleep, people who slept less had higher interleukin-6 (IL-6) and tumor necrosis factor (TNF) production after a marital problem discussion, but adaptive emotional regulation strategies protected who slept less from inflammatory reactivity (189). Furthermore, linkage between autonomic fluctuations measured as HRV synchrony appears to capture engagement with, or an inability to disengage from, a hostile exchange among married couples (190). Stronger HRV synchrony related to situational factors during conflict predicted greater negative affect reactivity, framing conflict as a novel social-biological pathway to inflammation-related diseases.

Emotional regulation and inflammation was modeled as bidirectional pathways linking peripheral inflammation and neural circuitries serving emotional processing and regulation (191). In this model, levels of peripheral inflammation and resting state functional connectivity (rsFC) within the emotional regulation and central executive network are co-regulated. Relationships between inflammation (CRP, IL-6, IL-10, TNF) and rsFC (measured by fMRI) involved in immune-to-brain signaling were found. Key neurobiological correlates of emotional regulation strategies and their effects on mental and physical health include the sub-regions of prefrontal cortex that play a key regulatory role in autonomic, endocrine, and immunological processes. These effects lead to a novel neuro-immuno-affective framework that targets improving emotional regulation, in order to: (1) reduce negative effects associated with depression and/or anxiety; and (2) alter endocrine and immune responses (e.g., reduce inflammation) with changes in activity within (and connectivity between) brain systems that support (successful) emotional regulation (186). Such a framework may be adapted for psychiatric treatment protocols that holistically incorporate neural and immunological biomarkers to promote mental and physical health.

### Emotional Regulation and Depression and Anxiety

For decades, emotional regulation has been thought to be an essential, if underemphasized, feature of mental health (192). Emotional regulation is a factor in mood disorders and emotional dysregulation is prominent in depression and anxiety. People with affective disorders have difficulties implementing the adaptive strategies that are commonly deployed by normal emotional regulation because depression and anxiety associated with various mood and affective disorders negatively impact cognitive processes involved in emotional regulation (193). Depression is understood to be a disorder of emotional regulation (194). In a study of the relationship between depression vulnerability and difficulties with emotional regulation in groups of recovered-depressed and never-depressed participants, emotional suppression was found to be ineffective for down-regulating negative emotions, providing evidence for a role for endogenous emotional regulation but not suppression in depression vulnerability (195). Biological and psychological vulnerabilities associated with anxiety produce increased emotional reactivity, attentional biases toward threat, global tendencies to experience emotions as aversive, and to engage in avoidant processing and behavior (196). In a meta-analysis of Japanese individuals, poor emotional regulation correlated with both depression and anxiety, whereas good emotional regulation had significantly negative correlation with both disorders (197). Meta-analysis of 94 published studies of measures of emotional regulation and well-being revealed relationships in the expected direction (198). These results showed poor emotional regulation strategies had a moderate negative correlation with well-being whereas good emotional regulation strategies had a moderate positive correlation with well-being. Thus, it appears to be important to improve emotional regulation when aiming to improve well-being in people (199) and patients with chronic pain (179, 180), inflammation (200), and stress-related (183), and mental disorders (201–203) (Figure 3).

## Putative Pathways of Effects of MM on Cardiac Autonomic Control of HRV, Emotional Regulation, and Well-Being

Physical (such as pain), social, psychological, and environmental stressors cause dysfunctional homeostatic regulation of autonomic cardiac control leading the individual to shift into a state of persistent sympathetic activation and lowered HRV (74). We propose that MM exerts a directional effect on mood and restores autonomic homeostasis *via* HRV. This shift provides the individual with top-down control of mood management and strengthens emotional regulation, thereby reducing anxiety, depression, inflammation, and sensitized pain. In this article, we seek to better understand MM by considering its mechanistic underpinnings, based on the overarching hypothesis that treatment with MM acts *via* the vagus nerve to enhance mood, HRV, and emotional self-regulation and reduces sympathetic overactivation, physiologic stress, and inflammation. A better understanding of these mechanisms of MM will facilitate broader application and achievement of greater clinical benefits of MM by seeing how to reduce causative factors leading to mood, pain, and inflammation in clinical settings while at the same time developing, deploying, and improving therapeutic strategies to control autonomic dysfunction.

In our hypothesized model, autonomic control centers in the brainstem bi-directionally influence and are influenced by the cerebral circuits that modulate emotional regulation (depression, anxiety), inflammation, and pain. Cortical higher centers modify the activity of the medullary centers and are particularly important in stimulating CV responses to emotions and stress (44). The literature shows that MM affects HRV in predictable ways. It is not surprising that several studies have established that music affects HRV both in healthy individuals (169–171, 204–210) and patients (211–224). It is also not surprising that the relationship between music and HRV is a complex one; for example, exciting music has the effect of decreasing HF HRV power, which is associated with PNS activation (49) as compared to tranquilizing music (225).

## Effect Size Calculation of PMM on HRV

As a preliminary test of our hypothesis that MM affects HRV in a clinical (including peri-operative surgical) setting, we performed an effect size calculation. Forty-three published articles were identified as potentially having data that could be used in calculation of effect sizes for effect size of music on HRV in the perioperative surgical settings. Of these, 22 articles reported data that could be used in a calculation of effect size. These studies were selected using two criteria: (1) Independent groups or mixed random assignment design with between-subject data (no non-independent i.e. within-subject data) and (2) tabulated data including group standard deviations (no graphs) were available. All comparisons used a With MM vs. No MM comparison. Comparisons used post-music intervention group means (not pre-post within group changes). Studies were sorted for Independent groups or Mixed Design with random assignment vs. non-independent data and population type. The ratio of LF power to HF power was selected as the variable to be used for determination of effect size because LF/HF ratio is a stable indicator of relative sympathetic to parasympathetic activation under resting conditions and normal respiration. Low frequency is associated with SNS output while HF is associated with PNS output. Thus, a lower LF/HF ratio indicates a relatively higher proportion of PNS output. Six studies using patient samples were included in the effect size analysis. Six published studies met these criteria (211, 212, 217, 220, 221, 226). Mean LF/HF ratios were: 1.68 (0.48) for With MM and 2.44 (0.65) for No MM. Independent sample *t*-test (equal variance shown by Levene Test) was significant [*t*_(10)_ = −2.29, *p* (one-tailed) = 0.023]. The average unweighted effect size estimate was −0.39 (With MM < No MM).

## Hypothesis and Clinical-Experimental Research

Our central hypothesis is that activation of efferent myelinated ventral vagal cholinergic output onto the heart is critical for the efficacy of PMM, and MM in general. The resulting strengthening of PNS activity reduces stress responding. The process can be indexed non-invasively by HRV and experimentally demonstrated experimentally by vagotomy.

Surgical trauma induces physiologic stress and dysfunction of the ANS and its modulation of stress and immune responses to surgical trauma (227, 228). The mechanism of PMM may be to reduce post-surgical morbidity and improve surgical outcomes by decreasing sustained sympathetic activation and preserving metabolic and immune homeostasis (26, 229). We propose to test this hypothesis with a research team that possesses collective clinical and experimental expertise in perioperative settings and experimental animal models. Thus, examining the effects of surgery on autonomic, stress, immune, and mood (depression and anxiety) dysfunction, as well as opioid use and recovery parameters becomes feasible. The long-term goal of this line of research is to maximize clinical benefits of MM by analyzing its mechanism of action. There is a critical need to determine the mechanisms of PMM to maximize its efficacy and broaden the field of application of allied interventions that share the same general mechanism of action that can be deployed into the clinical setting.

## Discussion

The significance of PMM in the surgical contemporary health care system has greater relevance because research on the cognitive and affective pathways of PMM is occurring throughout the Translational Continuum, from preclinical lab studies through implementation in outpatient clinics. Evidence indicates that music affects the ANS, stimulating PNS activity and decreasing postoperative pain and analgesic use even in patients under general anesthesia. Perioperative Music Medicine implemented at the point-of-care is quite feasible and low-cost. However, further research is warranted to elucidate the neural pathways of ANS responding to PMM and whether PMM reduces psychophysiological stress associated with surgery. The model of PMM is supported by empirical data and the effect size calculation of PMM on HRV is significant.

Beyond the surgical suites, MM can be implemented broadly throughout the health care system either by self-administration or with the aid of a music therapist. The impact of chronic pain and chronic stress (due to general stressors in addition to pain) is to lower HRV indices. Chronic pain, chronic stress, and HRV are inter-related and strongly associated with the body's response of inflammation, depression, and anxiety. Heart Rate Variability can be increased and stress responding lowered by a number of interventions other than MM. The common factor underlying the benefit of MM and all the interventions that increase HRV, also variously called “increased resilience” or “well-being,” appears to be due to a strengthening of emotional regulation and includes interaction between elements of emotional regulation, chronic pain, chronic stress, inflammation, depression, and anxiety.

A central pathway of the mechanism of ANS response to psychophysiological stress is autonomic control of HRV, reflecting restoration of sympathovagal function from the states of hyper-arousal caused by chronic pain and stress. Vagal afference plays an important role in signaling to cortical and brainstem systems regulating physiological homeostasis. Increasing the level of an individual's HRV stimulates activity in the emotional regulation neural network, while decreased HRV is associated with loss of emotional regulation.

Experimental animal models allow deconstruction of MM into its component mechanisms. Strategies to understand the sensory and physiological underpinnings of MM effects have uncovered the important contributions of music preference and sound perception. Some data have shown that gender plays a role in animal responses to music, and that music may improve task performance in rodents and non-human primates. The brain regions involved in MM responding are being identified. Lowered stress responding due to MM is indicated by reduced sympathetic and increased parasympathetic activity, and reduced blood pressure and serum corticosterone levels in both normal and hypertensive rodents. Results from studies of a variety of experimentally-induced pathophysiological and disease conditions are consistent with the conclusion that music appears to be an effective treatment.

Surgical procedures are associated with significant pain, which is routinely treated with opioid medications. However, opioids have multiple detrimental side effects, delay recovery, and contribute to complications to postoperative mortality and morbidity. A primary aim of future research will be to conduct a RCT powered based on anticipated effects of PMM on HRV. A related aim of clinical PMM research must be to analyze the associations between HRV and stress and inflammatory markers, and patient-reported outcomes of opioid use, pain, depression, and anxiety.

However, another and equally important aim will be to determine the neuronal networks mediating the mechanism of music modulation of peripheral physiology in experimental animal orthopedic surgery. Functional analyses using pharmacologic, surgical, and genetic approaches to examine how music modifies activity of pathways of neuro-modulation: the HPA and corticosteroids, sympathetic catecholamines, and parasympathetic vagal and cholinergic system in an experimental model of orthopedic surgery. In this protocol, surgical vagotomy and pharmacologic blockade plus genetic knock-out of cholinergic receptors are hypothesized to eliminate the role of the vagus in PMM responding and prevent reduction of inflammation after orthopedic surgery.

The rationale for the need of an animal model in parallel to clinical trials of PMM is compelling. The clinical and animal model aims are interdependent. Surgical patients exposed to PMM may have a range of physiological responses depending on how much they are engaged with the music vs. engaged with everything else going on in the environment. The clinical research model will use HRV and various inflammation biomarkers and psychological instruments to detect how much PMM is working in humans. The HRV response is a way to measure the degree of psychophysiological response. Experimental animal models can mimic surgical trauma and undergo selective neurectomies, genetic, and pharmacological inhibition of the hypothesized neuronal pathways involved in PMM (230, 231). This level of experimental control is only possible in experimental animals. Thus, experimental animal surgery can confirm the hypothesized mechanism of the human model by using specific neural and humoral interruptions. These mechanistic studies will be critical to define the potential limitations of MM in specific cohorts of patients and how to improve the treatments.

## Author Contributions

All authors listed have made a substantial, direct, and intellectual contribution to the work and approved it for publication.

## Conflict of Interest

The authors declare that the research was conducted in the absence of any commercial or financial relationships that could be construed as a potential conflict of interest.

## Publisher's Note

All claims expressed in this article are solely those of the authors and do not necessarily represent those of their affiliated organizations, or those of the publisher, the editors and the reviewers. Any product that may be evaluated in this article, or claim that may be made by its manufacturer, is not guaranteed or endorsed by the publisher.
